# Hospital factors and patient characteristics in the treatment of colorectal cancer: a population based study

**DOI:** 10.1186/1471-2458-12-775

**Published:** 2012-09-12

**Authors:** Carlotta Sacerdote, Ileana Baldi, Oscar Bertetto, Daniela DiCuonzo, Enzo Farina, Eva Pagano, Rosalba Rosato, Carlo Senore, Franco Merletti, Giovannino Ciccone

**Affiliations:** 1 Cancer Epidemiology Unit, San Giovanni Battista Hospital, CPO Piemonte and University of Turin, Via Santena 7, 10129, Torino, Italy; 2 Medical Oncology Unit, San Giovanni Battista Hospital, Corso Bramante 88, Turin, Italy; 3 General Surgery Unit, San Giovanni Battista Hospital, Corso Bramante 88, Turin, Italy; 4 Department of Psychology, University of Turin, Turin, Italy; 5 Cancer Epidemiology Unit, San Giovanni Battista Hospital, CPO Piemonte, Via S Francesco da Paola 31, Turin, Italy

**Keywords:** Colorectal cancer, Quality of care, Radiotherapy, In-hospital mortality, Hospital discharges

## Abstract

**Background:**

The present study focuses on the analysis of social, clinical and hospital characteristics that can lead to disparities in the management and outcome of care. To that end, indicators of the quality of initial treatment delivered to newly-diagnosed colorectal cancer patients in a North-Western Region of Italy, were investigated using administrative data.

**Methods:**

The cohort includes all incident colorectal cancer patients (*N* = 24,187) selected by a validated algorithm from the Piedmont Hospital Discharge Record system over an 8-year period (2000–2007).

Three indicators of quality of care in this population-based cohort were evaluated: the proportion of preoperative radiotherapy (RT) and of abdominoperineal (AP) resection in rectal cancer patients, and the proportion of postoperative in-hospital mortality in colorectal cancer patients.

**Results:**

Among rectal cancers, older patients were less likely to have preoperative RT, and more likely to receive an AP resection compared to younger patients. The probability of undergoing preoperative RT and AP resection was reduced in females compared to males (odds ratio (OR) 0.77, 95% confidence interval (CI) 0.64-0.93 and OR 0.78, 95%CI 0.69-0.89, respectively). However, there was a trend of increasing RT over time (p for trend <0.01). The probability of undergoing AP resection was increased in less-educated patients and in hospitals with a low caseload.

A higher risk of postoperative in-hospital mortality was found among colorectal cancer patients who were older, male, (female versus male OR 0.71, 95%CI 0.60-0.84), unmarried (OR 1.32, 95%CI 1.09-1.59) or with unknown marital status.

**Conclusions:**

The study provides evidence of the importance of social, clinical and hospital characteristics on the equity and quality of care in a Southern European country with an open-access public health care system.

## Background

Colorectal cancer is a common malignancy and its incidence is increasing in industrialized countries [1]. While there are several studies on new drugs for colorectal cancer [2-4], fewer studies have been conducted to assess the appropriateness and equity of care provided to colorectal cancer patients at a population level [5-7].

Administrative data provide information on quality of care, and monitor indicators of care that can be used for assessment at a population- and hospital-level [8,9]. The linkage of different administrative sources provides an efficient method for gathering data on individual patterns of care [10]. Although clinical data available in administrative databases are considered limited in accuracy [11], their validity should be specifically assessed, as variations exist not only between countries and periods, but also between variables included. The use of administrative data in the assessment of quality of care among colorectal cancer patients is common in the United States [12,13], Canada [14] and Northern Europe (United Kingdom, Sweden and Denmark) [6,7,15]. To our knowledge, there are no published population-based studies on the quality of care among colorectal cancer patients in Southern Europe.

Preoperative radiotherapy (RT) in stage II or III rectal cancer has been recommended by regional guidelines since 2001 [16] and recently confirmed [17] to aid in the reduction of local recurrences and tumor shrinkage before surgery. In the last 10 years in Europe, total mesorectal excision with sphincter-sparing procedure is the preferred choice over sphincter-ablating procedures. The shift toward sphincter-sparing procedures with the preservation of normal bowel activity is the result of several studies that have indicated similar outcomes [12] and improved quality of life [13,18-20]. An abdominoperineal (AP) resection, which denotes permanent colostomy, is unavoidable in some circumstances. However, surgeons with a higher caseload of rectal cancer patients, have been shown to perform a lower proportion of AP resections and have patients with better survival [14,15,21-23]. Finally postoperative in-hospital mortality has frequently been used as a measure of quality of care, but careful risk adjustment is needed to minimize the role of unbalanced case-mix distribution between providers [24]. All these indicators have been used in several studies that analyzed hospital statistics [7,14,25].

The present study focuses on the analysis of non-clinical factors that can lead to disparities in the management and outcome of care. To that end, social, clinical and hospital determinants of the quality of initial treatment delivered to newly-diagnosed colorectal cancer patients who underwent a surgical procedure between 2000 and 2007 in the Piedmont Region (North-Western Italy) were investigated, using routinely available administrative data.

## Methods

### Study population

The study cohort of incident colorectal cancer cases in the resident population of the Piedmont Region (about 4.3 million inhabitants) was identified from the Piedmont Hospital Discharge Record (HDR) system over an 8-year period using a validated algorithm [26] based on combinations of diagnostic and surgical procedure codes according to the International Classification of Diseases, 9th Revision, Clinical Modification (ICD-9-CM). The HDR system routinely collects both inpatients and day-care activity from all regional private and public hospitals publicly funded. The Italian National Health Service covers the entire population and private funding is residual, particularly for life-threatening diseases like cancer. Providers, both public and private, including hospitals outside the region, to be reimbursed by the NHS need to deliver a Hospital Discharge Record. As a consequence, all the in-patient and day-care activities are included in the database for administrative purpose. Patient’s HDRs are identified by means of an encrypted unique identification code based on the tax identification number. Multiple records relative to each patient are linked by means of this encrypted code.

Among all patients discharged between January 1, 2000 and December 31, 2007 with a surgical diagnosis-related group (DRG) claim, those with a diagnosis of malignant neoplasm of the colon (ICD-9-CM: 153.0-153.9) or malignant neoplasm of the rectum or rectosigmoid junction (ICD-9-CM: 154.0-154.1, 154.8) were selected (*N* = 29,248). Of this cohort, patients with potentially prevalent cancer (those who had been hospitalized at any time during the previous 5 years with either colon or rectal cancer), as well as those with a history of colorectal cancer (ICD-9-CM V10.05-V10.06), were excluded (N = 3,946).

**Figure 1:**
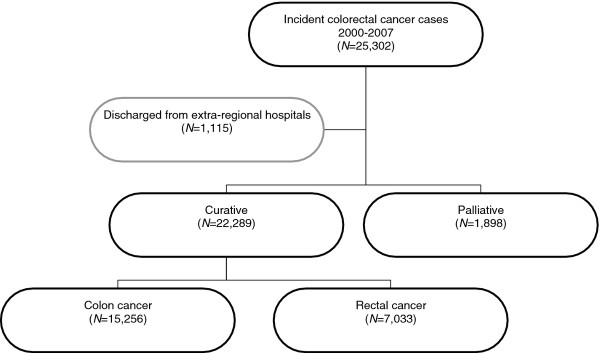
Flow chart of inclusion of colorectal cancer patients into the cohort.

Of the remaining 25,302 incident colorectal cancer cases, an additional 1,115 cases (4.4%) that were discharged from extra-regional hospitals were excluded (Figure 1). Patients were classified as colon cancer and rectal cancer patients on the basis of the site of the tumors. The 142 patients with a double lesion in colon and rectum were classified as rectal cancer patients.

### Patient characteristics

The HDR system routinely includes patient’s demographic data, admission and discharge dates, admission referral source, discharge status, up to six ICD-9-CM discharge diagnostic and procedure codes, the regional code of the facility, the diagnosis-related group and its tariff.

Firstly, the surgical approach was classified as palliative or curative; this last group was then split according to the surgical procedure performed: AP resection (ICD9: 48.5, 48.62) or other resections (ICD9: 45.4x-45.8x, 48.3x-48.4x, 48.61, 48.63-48.69). Cases were classified as having concomitant obstruction (ICD-9-CM 560.9), perforation (ICD-9-CM 569.83), or an emergency admission (OPE) or not.

Using all the diagnosis codes from the first surgical admission, disease staging [27] was used to classify patients into two broad categories, according to the absence or presence of loco-regional or distant metastases. To maximize ascertainment of comorbidity, all hospital discharges that occurred within 12 months before the indexed surgical admission were identified, and all the coded diagnoses used to calculate comorbidity by the Charlson index (hereafter referred to as comorbidity), as adapted by Romano et al. for use with claims data [28].

Additionally, for each patient, the risk-of-mortality score was calculated using the 3 M all-patient-refined (APR)-DRG classification system, V20 [29] classified in the categories low, medium, high and extreme. This proprietary software package assigns a value from 1 to 4 to each patient in each APR-DRG group, corresponding to increasing risk of death. This score is generated using data on age, the presence of comorbidities, and some procedures and their interactions, thereby allowing a meaningful risk adjustment tool for outcome analyses.

Hospital volume was classified into three classes according to annual caseload of colorectal surgical resection procedures (≤10, 11–25 and >25 for rectal cancer and <25, 26–60 and >60 for colon cancer), based on categories presented in the recommendations of the regional guidelines [16]. Hospitals were also categorized according to the presence of a RT service.

For each patient, accessibility to preoperative RT was measured as the distance between their residence and the nearest RT service, by car and under normal traffic conditions, [30]. Three categories were defined: same city or less than 15 minutes; 15 to less than 30 minutes; 30 minutes or more.

### Outcomes

Three indicators of quality of care were selected: the proportions of rectal cancer patients who received preoperative RT or abdominoperineal (AP) resection, and the proportion of all colorectal cancer patients who died post-operatively in hospital.

### Statistical analysis

Random-intercept logistic regression models were used to analyze data, accounting for within-hospital outcome correlation. The logistic models included patients’ age (≤60, 61–70, 71–80 or >80 years old), gender, education level (categorized as ‘secondary or more’, ‘intermediate’, ‘primary’ or ‘unknown’), marital status (classified as ‘married’, ‘unmarried’: single, separated, divorced, or widowed, and ‘unknown’) and year of admission. Given the strong correlation between APR-DRG risk-of-mortality score, tumor stage and comorbidity (Charlson index 0, >1), these predictors were not included in the models simultaneously.

The results are expressed as odds ratios (OR) and 95% confidence intervals (95%CI). Statistical analyses were performed using STATA (version 9.2) software.

### Validation

In this study the method used to identify incident colorectal cancer cases included in the study cohort sample was validated first, followed by the indicators obtained from the HDR system.

The algorithm adopted to identify colorectal cancer cases in this study cohort has been previously validated using the data of the Piedmont Cancer Registry as a reference standard (positive predictive value of the identification algorithm was 89.9%, 95%CI 87.3-92.1) [26].

The accuracy of key variables obtained from the HDR system was further validated using a sample of 605 patients randomly selected from the same study population as a reference standard (aged 50–69, surgically treated during 2001–2002). In this high-resolution sample, for each patient we abstracted and analyzed all clinical records of surgical, and RT hospitalization identified from the HDR system (2001–2005), and found 100% reproducibility for the variables “emergency admission” and “mortality”. Furthermore 96% of the colorectal cancers (n = 580) are histopathologically confirmed. Table 1 shows the validation data for “type of cancer”, “type of surgery” and “presence of metastasis”.

**Table 1:** Validation results based on a survey of 605 clinical records (the high-resolution sample)

	**High-resolution sample (gold standard)**					
			**Colon**	**Rectum**	**Missing**	**TOTAL**
		**Colon**	**181**	6	0	187
	**Type of cancer**	**Rectum**	28	**390**	0	418
		**TOTAL**	209	396	0	**605**
			**Curative intent**	**Palliative intent**	**Missing**	**TOTAL**
		**Curative intent**	**567**	32	0	599
	**Type of surgery**	**Palliative intent**	5	**1**	0	6
		**TOTAL**	572	33	0	**605**
**Study cohort sample**			**AP resection**	**Minor resection**	**Missing**	**TOTAL**
		**AP resection**	**78**	11	0	89
	**Surgery for rectal cancer**	**Minor resection**	15	**281**	**5**	301
		**Missing**	0	0	0	**0**
		**TOTAL**	93	292	5	**390**
			**Yes**	**No**	**Missing**	
		**Yes**	**65**	2	0	65
	**Presence of metastasis**	**No**	25	**509**	**6**	540
		**Missing**	0	0	**0**	0
		**TOTAL**	90	511	6	**605**

AP: abdominoperineal.

### Ethical issues

The study is a retrospective clinical audit to monitor the quality of treatment, delivered to colorectal cancer patients. It has been conducted as recommended in the regional guidelines [16], by the Centro di Riferimento per l'Epidemiologia e la Prevenzione Oncologica in Piemonte under the mandate of the regional health authority, using preexisting encrypted administrative data. For these reasons this project was exempt from ethical review.

## Results

During the 8-year period, 25,302 patients with incident colorectal cancer were identified: 24,187 were treated in the Piedmont Region and 1,115 (4.4%) in extra-regional hospitals.

Figure 1 depicts the flow-chart of the inclusion of colorectal cancer patients in the cohort: 22,289 (92%) potentially curative resections were identified in 15,256 and 7,033 colon and rectal cancer patients respectively.

Among the 5,437 rectal cancer patients who underwent an elective intervention, 727 (13.4%) underwent preoperative RT (Table 2). Older patients were less likely to have RT before surgery. The probability to undergoing RT was also reduced in females versus males (OR 0.77, 95%CI 0.64-0.93), and, as expected, for patients with comorbidity (Charlson index ≥1 OR 0.73, 95%CI 0.59-0.90) or metastases (0.47, 95%CI 0.35-0.65). Furthermore, there was an unexpected effect of educational level: less-educated patients had a higher probability of receiving neoadjuvant RT. There was a clear trend of increasing RT over time (*P* for trend <0.01). Patients were more likely to receive RT if the hospital where the surgery was performed had a RT service (OR 2.24, 95%CI 1.77-2.85). Almost 47% of the sample lived in a city that had a RT service, and about 16% of patients lived 30 minutes or more away from a RT service. The adjusted OR to receive neoadjuvant RT tended to decrease with increasing distance between a patient’s residence and RT service (P for trend = 0.06). We performed a sensitivity analysis, including in the model the hospital volume instead of RT service. The results of this model are similar for all variables and the adjusted OR to receive neoadjuvant RT decreased with decreasing of the hospital volume lower than 25 case at year (11–25 cases/year versus >25 cases/year OR 0.84, 95%CI 0.41-0.98).

**Table 2:** Preoperative radiotherapy (RT) (followed by planned surgical admission) in 5,437 incident rectal cancer patients

	**Total (*N*=5437)**	**Preoperative RT (*N*=727)**	Random-effect model*		
	**No.**	**%**	**OR**	**95% CI**	**P**
**Age (years)**					
≤60	1,082	19.2	1		<0.001
61-70	1,707	14.8	0.71	(0.57-0.90)	
71-80	1,958	11.4	0.51	(0.40-0.65)	
>80	690	6.1	0.24	(0.16-0.35)	
**Gender**					
Male	3,375	14.2	1		0.03
Female	2,062	12.1	0.77	(0.64-0.93)	
**Educational level**					
Secondary or more	813	13.9	1		<0.001
Intermediate	1,244	15.3	1.35	(1.03-1.77)	
Primary	2,869	11.7	1.41	(1.09-1.83)	
Unknown	511	17.4	1.16	(0.79-1.71)	
**Marital status**^†^					
Married	3,720	13.3	1		<0.001
Unmarried	1,352	11.5	0.99	(0.80-1.23)	
Unknown	365	20.8	0.84	(0.58-1.20)	
**Disease staging**					
Absence of metastases	4,836	13.9	1		0.01
Presence of metastases	601	8.8	0.47	(0.35-0.65)	
**Comorbidity (Charlson index)**					
0	4,068	14.1	1		0.01
≥1	1,369	11.2	0.73	(0.59-0.90)	
**Year of admission**					
2000	654	6.9	1		<0.001
2001	706	9.6	1.51	(1.00-2.29)	
2002	617	12.3	1.96	(1.30-2.95)	
2003	607	15.3	2.76	(1.85-4.11)	
2004	729	16.0	2.98	(2.02-4.40)	
2005	691	16.5	2.90	(1.96-4.29)	
2006	738	15.2	2.82	(1.90-4.17)	
2007	695	14.7	3.04	(2.03-4.56)	
**RT service**					
Absent	2,775	9.4	1		<0.001
Present	2,662	17.5	2.24	(1.77-2.85)	
**Distance to RT service (min.)**					
Same city or <15’	2,576	15.6	1		<0.001
15’ to <30’	1,984	12.2	0.97	(0.80-1.18)	
≥30’	877	9.6	0.82	(0.60-1.12)	
**Hospital volume (annual caseload)**					
>25	3,076	17.6	-	*	<0.001
11-25	1,778	6.8	-	*	
≤10	583	11.1	-	*	

*Given the correlation between hospital volume and presence of RT service, these predictors were not included in the models simultaneously.

^†^Unmarried includes single, separated, divorced and widowed.

OR: odds ratio, CI: confidence interval, APR-DRG: all-patient-refined-diagnosis-related group, RT: radiotherapy.

Table 3 shows the proportion of patients receiving AP resection versus other, more conservative resections, among 7,033 rectal cancer patients, for each variable in the model. Patients older than 70 years were more likely to receive an AP resection compared to patients aged 60 years or younger. The probability of receiving AP resection also increased in less-educated patients and in hospitals with a low volume. AP resection was performed less frequently in women (OR 0.78, 95%CI 0.69-0.89). There was no independent association between disease stage, Charlson index, year of admission or emergency admission and having an AP resection for rectal cancer.

**Table 3:** Abdominoperineal (AP) resection* versus other resections^**†**^**(reference group) in 7,033 incident rectal cancer patients**

	**Total (*N*** **= 7033)**	**AP resection (N = 727)**	**Random-effect model**^‡^		
	**No.**	**%**	**OR**	**(95% CI)**	**P**
**Age (years)**					
≤60	1,342	17.4	1		<0.001
61-70	2,083	20.7	1.10	(0.91-1.33)	
71-80	2,536	23.9	1.30	(1.08-1.56)	
>80	1,072	24.6	1.38	(1.11-1.71)	
**Gender**					
Male	4,275	23.0	1		0.002
Female	2,758	19.9	0.78	(0.69-0.89)	
**Educational level**					
Secondary or more	969	17.7	1		<0.001
Intermediate	1,516	19.2	1.11	(0.90-1.38)	
Primary	3,796	23.9	1.40	(1.16-1.70)	
Unknown	752	22.1	1.29	(0.97-1.70)	
**Marital status**					
Married	4,651	21.3	1		0.002
Unmarried^§^	1,854	24.2	1.17	(1.02-1.35)	
Unknown	528	17.6	0.83	(0.63-1.10)	
**OPE**^‡^					
No	5,437	22.5	1		0.05
Yes	1,596	19.6	0.87	(0.75-1.01)	
**Disease staging**					
Absence of metastases	6,180	21.8	1		0.82
Presence of metastases	853	22.3	1.11	(0.93-1.32)	
**Comorbidity (Charlson index)**					
0	5,174	21.5	1		0.24
≥1	1,859	22.8	1.01	(0.89-1.16)	
**Year of admission**					
2000	861	23.0	1		0.04
2001	887	23.9	1.12	(0.89-1.41)	
2002	789	23.7	1.06	(0.84-1.35)	
2003	825	19.1	0.87	(0.68-1.11)	
2004	961	20.0	0.92	(0.73-1.16)	
2005	906	22.2	1.02	(0.80-1.28)	
2006	923	23.4	1.08	(0.86-1.36)	
2007	881	19.4	0.85	(0.67-1.09)	
**Hospital volume (annual caseload)**					
>25	3,688	20.6	1		0.001
11-25	2,560	22.1	1.03	(0.81-1.31)	
≤10	785	26.6	1.37	(1.03-1.82)	

*ICD9: 48.5, 48.62. ^†^ICD9: 45.4x-45.8x, 48.3x-48.4x, 48.61, 48.63-48.69.

^‡^Cases with obstruction or perforation or in emergency admission were classified in the OPE category.

^§^Unmarried includes single, separated, divorced and widowed.

OR: odds ratio, CI: confidence interval, APR-DRG: all-patient-refined-diagnosis-related group, OPE: obstruction, perforation or emergency admission.

The postoperative in-hospital mortality in 22,289 colorectal cancer patients is shown in Table 4. During the study period, 841 patients died in the hospital after a surgery with curative intent. The median length-of-stay in the hospital after surgery was 10 days (interquartile range 5 days) and 11 days (interquartile range 18 days) for deceased and alive colon cancer patients respectively, and 11 days (interquartile range 6 days) and 12 days (interquartile range 19 days) for deceased and alive colon cancer patients respectively. The odds of dying in hospital in older patients, notably the 71-80-year and over-80-year groups, was two-to-three times higher compared to patients aged 60 years or younger. Patients at higher risk of postoperative in-hospital mortality were male (females versus males OR 0.71, 95%CI 0.60-0.84), unmarried or with unknown marital status (OR 1.32, 95%CI 1.09-1.59 and OR 1.65, 95%CI 1.20-2.29 respectively), and with an emergency admission (OR 1.54, 95%CI 1.29-1.85). Furthermore, there was a trend of decreasing mortality risk over time. After adjustment for other variables, hospital volume, as measured by annual caseload, did not show any effect on mortality.

**Table 4:** Postoperative in-hospital mortality in 22289 incident colorectal cancer patients after curative surgery

	**Total (*N*** **= 22289)**	**Unadjusted mortality**	Random-effect model*		
	**No.**	**%**	**OR**	**95% CI**	**P**
**Age (years)**					
≤60	3,910	1.0	1		
61-70	6,224	1.9	1.76	(1.20-2.57)	<0.001
71-80	8,124	4.0	2.30	(1.61-3.28)	
>80	4,031	8.9	3.34	(2.33-4.79)	
**Gender**					
Male	12,309	4.0	1		0.36
Female	9,980	3.5	0.71	(0.60-0.84)	
**Educational level**					
Secondary or more	3,165	1.2	1		<0.001
Intermediate	4,745	2.6	1.14	(0.82-1.58)	
Primary	11,625	4.4	1.25	(0.95-1.67)	
Unknown	2,754	5.2	1.60	(1.10-2.32)	
**Marital status**					
Married	14,221	3.1	1		<0.001
Unmarried	6,212	5.0	1.32	(1.09-1.59)	
Unknown	1,856	5.5	1.65	(1.20-2.29)	
**OPE**					
No	14,558	1.8	1		<0.001
Yes	7,731	7.5	1.54	(1.29-1.85)	
**Disease staging**					
Absence of metastases	19,064	3.5	-		<0.001
Presence of metastases	3,225	5.7	-		
**Comorbidity (Charlson index)**					
0	16,145	3.2	-		<0.001
≥1	6,144	5.4	-		
**Year of admission**					
2000	2,458	4.1	1		0.55
2001	2,583	3.4	0.82	(0.59-1.14)	
2002	2,651	4.1	0.95	(0.69-1.29)	
2003	2,687	3.9	0.75	(0.55-1.03)	
2004	2,924	4.4	0.77	(0.57-1.05)	
2005	2,930	3.3	0.53	(0.38-0.73)	
2006	3,006	3.5	0.54	(0.39-0.74)	
2007	3,050	3.6	0.49	(0.34-0.68)	
**Tumor site**					
Colon	15,256	4.1	1		<0.001
Rectum	7,033	3.1	1.05	(0.87-1.27)	
**Hospital volume (annual caseload)**					
>60	9,371	3.5	1		0.10
26-60	9,430	3.9	0.87	(0.64-1.19)	
<25	3,488	4.3	1.14	(0.76-1.69)	
**APR-DRG risk-of-mortality score**					
Low	12,740	0.8	1		<0.001
Medium	7,869	3.9	4.47	(3.54-5.66)	
High	1,359	19.1	25.77	(19.80-33.54)	
Extreme	321	56.7	153.03	(109.81-213.27)	

*Given the correlation between APR-DRG risk-of-mortality score, tumor stage and comorbidity score these predictors were not included in the models simultaneously.

‡Unmarried includes single, separated, divorced and widowed.

OR: odds ratio; CI: confidence interval; APR-DRG: all-patient-refined-diagnosis-related group; OPE: obstruction, perforation or emergency admission.

## Discussion

This study focused on the relationship between social, clinical and hospital characteristics and postoperative in-hospital mortality after treatment for colorectal cancer. For rectal cancer patients only, the associations of such factors with the use of neoadjuvant RT and AP resection rates were also investigated.

In our population, older people with rectal cancer were less frequently treated with preoperative RT, were more likely to undergo AP resections and to die during hospitalization. A reason for the lower rate of preoperative RT may be that elderly patients were excluded from RT, either for medical reasons or as a consequence of difficult accessibility to RT facilities [31]. Previous studies suggested that elderly patients are usually diagnosed at advanced stages [32]. In addition, the older patients of the cohort present with a higher burden of comorbidities, which could also explain the lower probability to undergo a more conservative surgery, and the higher in-hospital mortality.

Male patients in our study underwent AP more often, had a higher in-hospital mortality and were more likely to have RT before rectal cancer surgery. Previous studies observed that survival after colorectal cancer resection is better in women than in men [33,34]. In particular, in a recent study in the United States, Paulson noted that women have a longer survival compared with men – despite the fact that they are more likely to have an emergency admission and at an older age – and they receive less aggressive medical treatment [35].

In the population of the Piedmont Region, marital status had a clear effect on in-hospital mortality, with unmarried patients and those with unknown marital status showing a significantly higher mortality risk than married patients. A possible explanation for this difference is diagnostic delay, which may affect the extent of disease at diagnosis, or differences in access to health care services [36]. However, a protective effect of family caregivers during hospitalization cannot be excluded.

Less-educated patients have a significantly higher probability to undergo preoperative RT. This is an unexpected finding, as less-educated people also have a higher risk to undergo AP resection and to die after colorectal surgery than more-educated people. Access to public health care services is provided free in Italy, and hospital admission is not clearly determined by social class [37]. Nevertheless, the higher rate of preoperative RT in less-educated patients may reflect a residual confounding by disease stage, with a lower proportion of early, screen-detected cases in this group (without indication for RT), as we already reported some years ago [38].

An extensive body of literature suggests that a hospital’s surgical volume of surgeries and hospital procedures is predictive of short- and long-term outcomes in patients undergoing complex medical and surgical procedures [22,39-42]. In our study, a hospital’s annual caseload was a predictor of the type of surgery performed among rectal cancer patients but not of in-hospital mortality. Finally, we found the presence of a RT service in the hospital where surgery was performed, and the distance between the patient’s residence and the nearest RT facility, to be two important predictive factors for preoperative RT. Most of these results, such as the role of comorbidity [43], hospital volume [39] and distance to RT facilities [42], have been reported by other studies, mostly from the United States. As such, this study has its added value in confirming the importance of these factors to the equity and quality of care in a Southern European country with an open-access public health care system.

The present study shares the limitations of research that uses routinely-collected data with respect of the completeness and accuracy of data coding and the use of limited clinical variables. In particular the lack of information on cancer stage could cause an underestimation of the proportion of incident rectal cancer that received the RT. Nevertheless, we performed an accurate validation of variables with the local cancer registry and a high-resolution clinical sample that confirmed the reliability of these administrative data. On the other hand, the major strength of our study is the population coverage and the availability of standardized data for a relatively long period.

## Conclusion

The study provides evidence of the importance of social, clinical and hospital patient characteristics on the equity and quality of care in a Southern European country with an open-access public health care system. Furthermore it offers an example of how these factors allow for the maintenance of a monitoring system that can be used to assess key indicators of process and outcome of initial treatment of colorectal cancer over time, with acceptable accuracy, at minimal cost.

## Abbreviations

RT: Preoperative radiotherapy; AP: Abdominoperineal; OR: Odds ratio; 95%CI: 95% confidence interval; HDR: Hospital discharge record; DRG: Diagnosis-related group; OPE: Obstruction perforation or emergency admission.

## Competing interests

All authors declared that they have no competing interests.

## Authors’ contributions

CS and GC conceived of the study and co-wrote the manuscript with contributions from all other authors; OB, EF. CS and FM contributed to study design, coordination of the study, acquisition of the data and their interpretation; IB, EP, RR and DC performed the statistical analysis and contributed to data interpretation; FM and GC conceived of the study, contributed to data interpretation, obtaining grant funding and financial support. All authors read and approved the final manuscript.

## Pre-publication history

The pre-publication history for this paper can be accessed here:

http://www.biomedcentral.com/1471-2458/12/775/prepub
